# A New Real-Time Cycle Slip Detection and Repair Method under High Ionospheric Activity for a Triple-Frequency GPS/BDS Receiver

**DOI:** 10.3390/s18020427

**Published:** 2018-02-01

**Authors:** Wanke Liu, Xueyuan Jin, Mingkui Wu, Jie Hu, Yun Wu

**Affiliations:** 1 School of Geodesy and Geomatics, Wuhan University, Wuhan 430079, China; wkliu@sgg.whu.edu.cn (W.L.); xueyuan.jin@whu.edu.cn (X.J.); jiehu1995@whu.edu.cn (J.H.); ywu@sgg.whu.edu.cn (Y.W.); 2 Collaborative Innovation Center for Geospatial Technology, Wuhan 430079, China; 3 Key Laboratory of Geophysical Geodesy, National Administration of Surveying, Mapping and Geoinformation, Wuhan 430079, China; 4 Faculty of Information Engineering, China University of Geosciences, Wuhan 430074, China

**Keywords:** GPS, BDS, triple-frequency, undifferenced observations, cycle slip, high ionospheric activity

## Abstract

Cycle slip detection and repair is a prerequisite for high-precision global navigation satellite system (GNSS)-based positioning. With the modernization and development of GNSS systems, more satellites are available to transmit triple-frequency signals, which allows the introduction of additional linear combinations and provides new opportunities for cycle slip detection and repair. In this paper, we present a new real-time cycle slip detection and repair method under high ionospheric activity for undifferenced Global Positioning System (GPS)/BeiDou Navigation Satellite System (BDS) triple-frequency observations collected with a single receiver. First, three optimal linearly independent geometry-free pseudorange minus phase combinations are selected to correctly and uniquely determine the cycle slips on the original triple-frequency carrier phase observations. Then, a second-order time-difference algorithm is employed for the pseudorange minus phase combinations to mitigate the impact of between-epoch ionospheric residuals on cycle slip detection, which is especially beneficial under high ionospheric activity. The performance of the approach is verified with static GPS/BDS triple-frequency observations that are collected with a 30 s sampling interval under active ionospheric conditions, and observations are manually inserted with simulated cycle slips. The results show that the method can correctly detect and repair cycle slips at a resolution as small as 1 cycle. Moreover, kinematic data collected from car-driven and airborne experiments are also processed to verify the performance of the method. The experimental results also demonstrate that the method is effective in processing kinematic data.

## 1. Introduction

The availability of high-quality carrier phase observations is a prerequisite for high-precision global navigation satellite system (GNSS)-based positioning. However, under conditions that cause signal interruption, a low signal-to-noise ratio or high receiver dynamics, GNSS receivers may lose track of navigation signals, and cycle slips occur, which result in discontinuities in the original carrier phase observations. Without careful consideration of such cycle slips, the precision and reliability of position and velocity estimates are seriously affected.

Over the past three decades, a number of cycle slip detection and repair methods have been developed and can be generally divided into three types according to the number of available signals. Polynomial fitting [1] and high-order between-epoch phase differentiation [2] can be implemented based on only single-frequency observations. However, they cannot detect small cycle slips due to the presence of clock error. Based on multi-constellation signals, Qian et al. [3] further proposed a cycle slip detection and repair method for a single-frequency Global Positioning System (GPS)/BeiDou Navigation Satellite System (BDS) receiver. But it is not applicable for single-system receiver because both GPS and BDS signals are required. Outlier detection methods, such as statistical hypothesis test (e.g., $\chi^{2}$ test) [4,5], robust estimation [6], the Bayesian method [7,8], and generalized likelihood ratio (CLR) test [9], can be utilized to detect and correct cycle slips as well. There are also many cycle slip detection and repair methods based on dual-frequency observations. Methods to detect and repair cycle slips using dual-frequency double-differenced or triple-differenced observations have been presented by Gao and Li [10], Bisnath and Langley [11], and Kim and Langley [12,13]. However, these methods require at least two receivers to realize the double-differenced or triple-differenced operation. To meet the requirement of high-precision positioning, such as Precise Point Positioning (PPP), it is more convenient and effective to implement cycle slip detection and repair based on undifferenced observations. The TurboEdit algorithm developed by Blewitt [14] is recognized as the first approach for undifferenced cycle slip detection and repair that uses dual-frequency observations. Both Melbourne–Wübbena (MW) linear combination [15,16,17] and ionospheric residual combination are used in TurboEdit. Although the TurboEdit algorithm is widely used, it has two disadvantages: First, the ionospheric residual combination is sensitive to variations in ionospheric delay residuals, which degrade the efficiency of the method. Furthermore, the wavelength of wide-lane (WL) combination is still relatively small, which also degrades the performance of the method when there is substantial pseudorange noise. de Lacy et al. [18] introduced the Bayesian criterion and polynomial fitting to detect cycle slips for dual-frequency observations with high sampling interval (e.g., 1 s or 5 s). At low sampling rate or under high ionospheric activity, their method is not suitable due to the large residuals produced by the ionospheric delay terms. Based on the TurboEdit algorithm, Liu [19] and Cai et al. [20] specially study cycle slip detection and repair under high ionospheric activity. However, their methods may not work well in situations with significant measurement noise. Based on a time-differenced model, the dual-frequency cycle slip detection and repair method has also been investigated by Zhang and Li [21], and Banville and Langley [22]. The external information such as precise orbit and clock corrections are required in these methods. There are also some cycle slip detection and repair methods that integrate the GNSS and other sensors. Aided by inertial navigation system (INS), cycle slip detection can be implemented with a single-frequency receiver [23,24] and dual-frequency receiver [25]. However, these methods are not flexible in application scenario due to the cost and complexity of GPS/INS.

With the modernization and development of GNSS systems, triple-frequency signals are now available, which allows the introduction of additional linear combinations with longer wavelengths, weaker ionospheric delays, and smaller noise [26,27]. The availability of triple-frequency observations provides new opportunities for cycle slip detection and repair. Dai et al. [28] proposed an instantaneous triple-frequency cycle slip detection and repair method that applies two geometry-free phase combinations with the least-squares ambiguity decorrelation adjustment (LAMBDA) [29] algorithm to search for cycle slip candidates. Since only two geometry-free phase combinations are used in the method, some undetectable cycle slips remain. Based on triple-frequency observations combination, Huang et al. [30], Li et al. [31], and de Lacy et al. [32] developed real-time cycle slip detection and repair methods. These methods assume that ionospheric delay varies smoothly over time. The performance of these methods might be reduced under high ionospheric activity. Zhao et al. [33], Yao et al. [34], and Li et al. [35] further proposed to compensate for the ionospheric bias by using ionospheric prediction. However, several epochs of continuous phase data that lack cycle slips are required to satisfy the criteria for the phase connection, e.g., 5 or 10 historic epochs are required in method presented by Li et al. [35]. Huang et al. [36] used three geometry-free and ionosphere-free (GIF) pseudorange minus phase combinations to eliminate the impact of ionospheric delay. Although GIF combinations are not affected by ionospheric delay, the cycle slip detection success rate of GIF combinations is decreased when there is significant pseudorange noise. On the other hand, Zhang [37] extended the geometry-based approach by integrating time-differenced pseudorange and carrier phase observations to estimate the integer number of triple-frequency cycle slips.

In this paper, we present a new real-time cycle slip detection and repair method under high ionospheric activity for undifferenced GPS/BDS triple-frequency observations collected with a single receiver. In this new approach, we jointly use three linearly independent geometry-free pseudorange minus phase combinations to efficiently detect and correctly repair the cycle slips on original triple-frequency carrier phase observations. Furthermore, a second-order time-difference algorithm is employed to mitigate the impact of between-epoch ionospheric variations, which improves the rounding success rate of estimated float cycle slips under high ionospheric activity. As observations of only three consecutive epochs (i.e., the current epoch and two previous epochs) are needed, cycle slips occurred at the current epoch can be efficiently detected and repaired in real time. Finally, we verify the performance of the approach by conducting static experiments during a magnetic storm and kinematic (car-driven and airborne) experiments. It is demonstrated that the presented method can detect and correctly repair cycle slips that are as small as 1 cycle in real time under high ionospheric activity. Compared with the existing double-frequency cycle slip detection and repair methods under high ionospheric activity [19,20], the impact of pseudorange noise will be reduced as the wavelength of triple-frequency combinations used in our proposed method is longer than that of dual-frequency WL combination. Compared with the triple-frequency cycle slip detection and repair methods utilizing ionospheric prediction [33,34,35], less consecutive epochs are required in our proposed method. Additionally, no external information is required in the proposed method with respect to the geometry-based time-differential positioning method, which requires precise orbit and clock correction [21,22].

The paper is organized as follows. In Section 2, we present the cycle slip detection and repair method based on geometry-free triple-frequency pseduorange minus phase combinations and second-order time-difference algorithm. In Section 3, we select optimal cycle slip detection and repair combinations for GPS and BDS, respectively. In Section 4, the proposed method is verified with real receiver data and simulated cycle slip, and the conclusions follow in Section 5.

## 2. Method of Triple-Frequency Cycle Slip Detection and Repair

The undifferenced pseudorange and carrier phase observation equations can be expressed as [38]:

(1) $$P_{i}=\rho +q_{i}I_{i}+\varepsilon_{P_{i}}$$

(2) $$\lambda_{i}\varphi_{i}=\rho -q_{i}I_{1}+\lambda_{i}N_{i}+\lambda_{i}\varepsilon_{\varphi_{i}}$$

where $P_{i}$ and $\varphi_{i}$ are pseudorange observations (in meters) and carrier phase observations (in cycles), respectively, on frequency $f_{i}$. ρ is the geometric distance from satellite to receiver, and includes the satellite and receiver clock errors, the tropospheric error, and the receiver and satellite hardware delays (in meters). $I_{1}$ is the first-order ionospheric delay at frequency $f_{1}$ (in meters) and it is multiplied by $q_{i}=f_{1}^{2}/f_{i}^{2}$ to obtain the corresponding delay at frequency $f_{i}$. $\lambda_{i}$ is the wavelength (in meters), $N_{i}$ is the integer ambiguity, and $\varepsilon_{P_{i}}$ and $\varepsilon_{\varphi_{i}}$ are pseudorange noise (in meters) and carrier phase observation noise (in cycles), respectively.

The geometry-based, linearly combined triple-frequency carrier phase observation equation is defined as [39]:

(3) $$\begin{array}{cc} \lambda_{ijk}\varphi_{ijk} & =\frac{if_{1}\lambda_{1}\varphi_{1}+jf_{2}\lambda_{2}\varphi_{2}+kf_{3}\lambda_{3}\varphi_{3}}{if_{1}+jf_{2}+kf_{3}}\\ & =\rho -\beta_{ijk}I_{1}+\lambda_{ijk}N_{ijk}+\lambda_{ijk}\varepsilon_{\varphi_{ijk}} \end{array}$$

where the combination coefficients i,j,k are integers. The linearly combined wavelength, integer ambiguity, and the ionospheric scale factor (ISF) are [39]:

$$\lambda_{ijk}=\frac{c}{if_{1}+jf_{2}+kf_{3}}$$

$$N_{ijk}=iN_{1}+jN_{2}+kN_{3}$$

$$\beta_{ijk}=\frac{f_{1}^{2}\left(i/f_{1}+j/f_{2}+k/f_{3}\right)}{if_{1}+jf_{2}+kf_{3}}$$

Similarly, the geometry-based, linearly combined triple-frequency pseudorange observation equation is defined as:

(4) $$\begin{array}{cc} P_{lmn} & =lP_{1}+mP_{2}+nP_{3}\\ & =\rho +\beta_{lmn}I_{1}+\varepsilon_{P_{lmn}} \end{array}$$

where the combination coefficients l,m,n are real numbers with a sum of 1 (i.e., l+m+n=1). $\beta_{lmn}=l+mf_{1}^{2}/f_{2}^{2}+nf_{1}^{2}/f_{3}^{2}$ is the ISF of $P_{lmn}$.

According to Equations (3) and (4), the geometry-free pseduorange minus phase combination can be expressed as:

(5) $$\varphi_{ijk}-\frac{P_{lmn}}{\lambda_{ijk}}=-K_{ijk,lmn}I_{1}+N_{ijk}+\varepsilon_{\varphi_{ijk}}-\varepsilon_{P_{lmn}}/\lambda_{ijk}$$

where $K_{ijk,lmn}=\left(\beta_{ijk}+\beta_{lmn}\right)/\lambda_{ijk}$. The geometry-free pseduorange minus phase combination can cancel out the geometric distance between satellite and receiver, the tropospheric delay, and satellite and receiver clock errors and hardware delays. Consequently, it is extremely suitable for cycle slip detection and repair for undifferenced observations under kinematic conditions.

The single-differenced observation equation for geometry-free pseudorange minus phase combinations between two consecutive epochs is defined as:

(6) $${\dot{\varphi }}_{ijk}-\frac{{\dot{P}}_{lmn}}{\lambda_{ijk}}=-K_{ijk,lmn}{\dot{I}}_{1}+{\dot{N}}_{ijk}+{\dot{\varepsilon }}_{\varphi_{ijk}}-{\dot{\varepsilon }}_{P_{lmn}}/\lambda_{ijk}$$

where the single dot denotes the first-order time-difference algorithm. The inter-frequency biases for both pseduorange and phase of both receiver and satellite are very stable such that they can be deemed as constant over a short time span. Moreover, the inter-observation-type bias (between pseudorange and phase) is stable as well. Consequently, the inter-frequency biases and inter-observation-type bias can be omitted through first-order time difference algorithm [35]. According to Equation (6), the first-order ionospheric delay variation (${\dot{I}}_{1}$) remains in the single-differenced observation of the geometry-free pseudorange minus phase combination. It can be too large to ignore under conditions of high ionospheric activity. The impact of the ionospheric variation is further discussed in Section 3. To further reduce the impact of the residual ionospheric error, the second-order time-difference algorithm can be employed.

The second-order, time-differenced, geometry-free, pseduorange minus phase combination observation equation is defined as:

(7) $${\ddot{\varphi }}_{ijk}-\frac{{\ddot{P}}_{lmn}}{\lambda_{ijk}}=-K_{ijk,lmn}{\ddot{I}}_{1}+{\ddot{N}}_{ijk}+{\ddot{\varepsilon }}_{\varphi_{ijk}}-{\ddot{\varepsilon }}_{P_{lmn}}/\lambda_{ijk}$$

where the double dot denotes the second-order time-difference algorithm. According to Equation (7), for three consecutive epochs t0, t1, and t2, ignoring small residual ionospheric error and noise term, the estimated combined cycle slip on the triple-frequency phase combination $\varphi_{ijk}$ at epoch t2, can be defined as:

(8) $$\begin{array}{c} {\hat{\dot{N}}}_{ijk}={\ddot{\varphi }}_{ijk}-\frac{{\ddot{P}}_{lmn}}{\lambda_{ijk}}\\ =\left[\varphi_{ijk\left(t2\right)}-\varphi_{ijk\left(t1\right)}-\left(\varphi_{ijk\left(t1\right)}-\varphi_{ijk\left(t0\right)}\right)\right]-\left[P_{lmn\left(t2\right)}-P_{lmn\left(t1\right)}-\left(P_{lmn\left(t1\right)}-P_{lmn\left(t0\right)}\right)\right]/\lambda_{ijk} \end{array}$$

Assuming that the noise terms of the carrier phase observations on each frequency are independent in time and are identical in variance (i.e., $\sigma_{\varphi 1}=\sigma_{\varphi 2}=\sigma_{\varphi 3}=\sigma_{\varphi }$), and the same is true for the pseudorange noise terms (i.e., $\sigma_{P1}=\sigma_{P2}=\sigma_{P3}=\sigma_{P}$), the standard deviations (STDs) of the estimated combined cycle slip ${\hat{\dot{N}}}_{ijk}$ is calculated as:

(9) $$\sigma_{{\hat{\dot{N}}}_{ijk}}=2\sqrt{\left(l^{2}+m^{2}+n^{2}\right)\sigma_{P}^{2}/\lambda_{ijk}^{2}+\left(i^{2}+j^{2}+k^{2}\right)\sigma_{\varphi }^{2}}$$

Assuming that ${\hat{\dot{N}}}_{ijk}$ is normally distributed, the cycle slip can be detected when the following condition is satisfied:

(10) $$\left|{\hat{\dot{\varphi }}}_{ijk}-\frac{{\hat{\dot{P}}}_{lmn}}{\lambda_{ijk}}\right|>s\sigma_{{\hat{\dot{N}}}_{ijk}}$$

where $s\sigma_{{\hat{\dot{N}}}_{ijk}}$ is the critical value for the cycle slip detection criterion, and the scalar s denotes the multiple of the standard deviation. s=3 and s=4 denote a 99.7% confidence level and a 99.9% confidence level, respectively.

To detect and repair cycle slips on the three original carrier phase observations, i.e., ${\dot{N}}_{1},{\dot{N}}_{2},{\dot{N}}_{3}$, three sets of linearly independent triple-frequency phase combinations are required.

Assuming the coefficients for three linear combinations are $\left(i_{1},j_{1},k_{1}\right)$, $\left(i_{2},j_{2},k_{2}\right)$, and $\left(i_{3},j_{3},k_{3}\right)$, the corresponding combined cycle slips are ${\dot{N}}_{i_{1}j_{1}k_{1}}$, ${\dot{N}}_{i_{2}j_{2}k_{2}}$, and ${\dot{N}}_{i_{3}j_{3}k_{3}}$, respectively. In such a case, the relationship between the combined cycle slip and the original cycle slips can be defined as:

(11) $$L=\left|\begin{array}{c} {\dot{N}}_{i_{1}j_{1}k_{1}}\\ {\dot{N}}_{i_{2}j_{2}k_{2}}\\ {\dot{N}}_{i_{3}j_{3}k_{3}} \end{array}\right|=\left|\begin{array}{ccc} i_{1} & j_{1} & k_{1}\\ i_{2} & j_{2} & k_{2}\\ i_{3} & j_{3} & k_{3} \end{array}\right|\left|\begin{array}{c} {\dot{N}}_{1}\\ {\dot{N}}_{2}\\ {\dot{N}}_{3} \end{array}\right|=Ax$$

According to Equation (11), in order to ensure that the cycle slips on the three original carrier phase observations can be recovered, the A-matrix must be reversible. In such a case, the cycle slips on the three original carrier phase observations can be obtained using $x=A^{-1}L$. As the combined cycle slip ${\dot{N}}_{ijk}$ is obtained by rounding the estimated combined cycle slip ${\hat{\dot{N}}}_{ijk}$, the elements of the L-matrix are all integers. To ensure that the cycle slips on the three original carrier phase observations that were computed with equation $x=A^{-1}L$ are also integers, the elements of the inverse of the A-matrix must be integers as well. This means that the A-matrix must satisfy the condition that the matrix elements are all integers and the determinant is equal to plus or minus 1 (i.e., det(A)=±1).

Generally, cycle slip validation is the step that follows the cycle slip determination. Similar to Equation (10), the cycle slip validation formula is:

(12) $$\left|{{\ddot{\varphi }}_{ijk}}^{repair}-\frac{{\ddot{P}}_{lmn}}{\lambda_{ijk}}\right|<s\sigma_{{\hat{\dot{N}}}_{ijk}}$$

where superscript “repair” indicates that the original carrier phase observations are already corrected by subtracting the integer cycle slips. If Equation (12) is not satisfied, the determined cycle slip is considered to be false.

## 3. Selection of Optimal Geometry-Free Pseudorange Minus Phase Combinations

According to Equation (8), the integral combined cycle slip ${\dot{N}}_{ijk}$ can be determined by rounding the estimated float combined cycle slip ${\hat{\dot{N}}}_{ijk}$. Consequently, a rounding success rate is introduced to evaluate the performance of the cycle slip detection.

Assuming that ${\hat{\dot{N}}}_{ijk}$ is normally distributed,

(13) $${\hat{\dot{N}}}_{ijk}∼\left({\dot{N}}_{ijk},\sigma_{{\hat{\dot{N}}}_{ijk}}^{2}\right)$$

where ${\dot{N}}_{ijk}$ is the true value of the combined cycle slip. Assuming that ${\overset{⌣}{\dot{N}}}_{ijk}$ is the nearest integer close to ${\hat{\dot{N}}}_{ijk}$ (i.e., ${\overset{⌣}{\dot{N}}}_{ijk}=\mathrm{int}\left({\hat{\dot{N}}}_{ijk}\right)$), the probability of ${\overset{⌣}{\dot{N}}}_{ijk}$ taking an integer i is [40]:

(14) $$P\left({\overset{⌣}{\dot{N}}}_{ijk}=i\right)=\int_{\left(i-{\dot{N}}_{ijk}\right)-0.5}^{\left(i-{\dot{N}}_{ijk}\right)+0.5}\frac{1}{\sqrt{2\pi }\sigma_{{\hat{\dot{N}}}_{ijk}}}\cdot \exp\left(-\frac{1}{2}z^{2}/\sigma_{{\hat{\dot{N}}}_{ijk}}^{2}\right)dz$$

**Figure 1:**
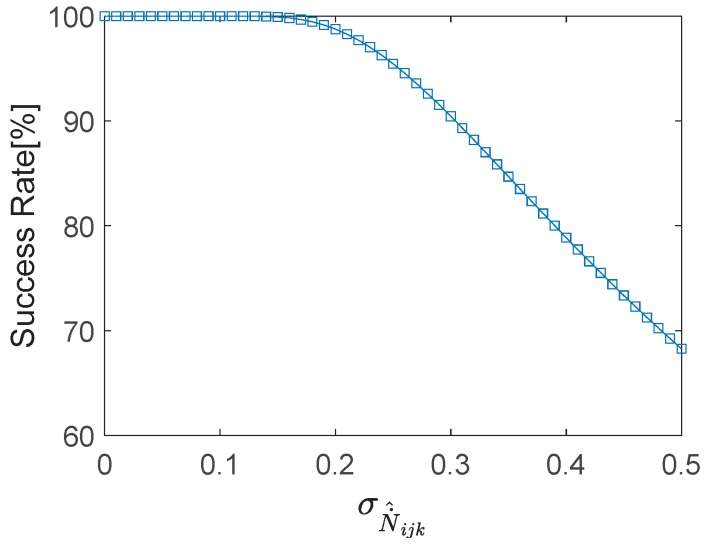
Relationship between rounding success rate and $\sigma_{{\hat{\dot{N}}}_{ijk}}$.

Based on this, the probability of obtaining the correct combined cycle slip ${\dot{N}}_{ijk}$ by rounding the estimated float combined cycle slip ${\hat{\dot{N}}}_{ijk}$ can be computed using [40]:

(15) $$\begin{array}{c} P\left({\overset{⌣}{\dot{N}}}_{ijk}={\dot{N}}_{ijk}\right)=P\left(\left|{\hat{\dot{N}}}_{ijk}-{\dot{N}}_{ijk}\right|\leq 0.5\right)=\\ 2\Phi \left(0.5\sigma_{{\hat{\dot{N}}}_{ijk}}^{-1}\right)-1 \end{array}$$

where $\Phi \left(x\right)=\int_{-\infty }^{x}\frac{1}{\sqrt{2\pi }}\exp\left(-\frac{1}{2}z^{2}\right)dz$. It is shown in Equation (15) that the probability of rounding to the correct combined cycle slip ${\dot{N}}_{ijk}$ increases as $\sigma_{{\hat{\dot{N}}}_{ijk}}$ (STD of ${\hat{\dot{N}}}_{ijk}$) decreases. The relationship between the rounding success rate and $\sigma_{{\hat{\dot{N}}}_{ijk}}$ is shown in Figure 1, and the rounding success rates when $\sigma_{{\hat{\dot{N}}}_{ijk}}=0.15,0.2,0.25$ (cycles) are listed in Table 1.

According to Equation (6), the residual ionospheric variation still remains in first-order time-difference pseudorange minus phase combination. Taking ionospheric variation into account, the bias-affected cycle slip rounding success rate can be computed by [41]:

(16) $$P_{bias}=\Phi \left(\frac{1+2bias_{ion}}{2\sigma_{{\hat{\dot{N}}}_{ijk}}}\right)+\Phi \left(\frac{1-2bias_{ion}}{2\sigma_{{\hat{\dot{N}}}_{ijk}}}\right)-1$$

where $bias_{ion}=-K_{ijk,lmn}{\dot{I}}_{1}$ and denotes ionospheric bias in cycles. When the between-epoch ionospheric variation ${\dot{I}}_{1}$ is larger than 0.02 m, the bias-affected cycle slip rounding success rate will be reduced to about 97% whereas it can be larger than 99% when ionospheric bias is so small that it can be ignored. The ionospheric total electron content (TEC) change rate can be over 0.03 TECU/s during ionosphere disturbances in the low latitude region [42,43]. In such cases, the impact of residual ionospheric variation in the first-order time-difference pseudorange and phase combination cannot be ignored. However, the second-order time-difference algorithm can further reduce the impact of ionospheric variation, resulting in improved cycle slip rounding success rate. 

According to Equations (7) and (10), the cycle slip detection criterion for the proposed second-order, time-difference, pseudorange minus phase combination method can be expressed as:

(17) $$\left|{\ddot{\varphi }}_{ijk}-{\ddot{P}}_{lmn}/\lambda_{ijk}+f\left(o\right)\right|>s\sigma_{{\hat{\dot{N}}}_{ijk}}$$

where $f\left(o\right)=-{\ddot{\varepsilon }}_{ijk}+{\ddot{\varepsilon }}_{lmn}/\lambda_{ijk}-K_{ijk,lmn}{\ddot{I}}_{1}$. 

To reduce the impact of f(o) and improve the rounding success rate, we propose the following three criteria for choosing the optimal pseudorange minus phase combinations: 

① It should have a relatively larger wavelength ($\lambda_{ijk}$) to reduce the impact of pseudorange noise.

② It should have a relatively smaller coefficient $K_{ijk,lmn}$ of ${\ddot{I}}_{1}$ to reduce the impact of the second-order, between-epoch, ionospheric delay variations. 

③ It should have relatively smaller $\sigma_{{\hat{\dot{N}}}_{ijk}}$ to ensure a higher rounding success rate for combined cycle slip.

For criterion ②, $K_{ijk,lmn}$ can be expressed as:

(18) $$\begin{array}{cc} K_{ijk,lmn} & =\left(\beta_{ijk}+\beta_{lmn}\right)/\lambda_{ijk}\\ & =\left(f_{1}/c+f_{1}\beta_{lmn}/c\right)\left[i+j\frac{f_{1}^{2}+f_{2}^{2}\beta_{lmn}}{f_{1}f_{2}+f_{1}f_{2}\beta_{lmn}}+k\frac{f_{1}^{2}+f_{3}^{2}\beta_{lmn}}{f_{1}f_{3}+f_{1}f_{3}\beta_{lmn}}\right] \end{array}$$

The $K_{ijk,lmn}$ of the geometry-free pseudorange minus phase combinations for BDS and GPS are 12.475(i+0.990j+0.987k) and 13.033(i+0.982j+0.986k), respectively. Generally, $K_{ijk,lmn}$ is small in cases where |i+j+k|≤2.

For criterion ③, according to Equation (9), pseudorange noise is the largest source of error in estimated combined cycle slips. To minimize the noise associated with estimated combined cycle slips (i.e., $\sigma_{{\hat{\dot{N}}}_{ijk}}=\min$), the combination coefficients l,m,n should be l=m=n=1/3, where $l^{2}+m^{2}+n^{2}=\min$ can be interpreted as the minimum distance from the origin point (0,0,0) to plane l+m+n=1.

The noise of pseudorange and carrier phase is about 1% of the code width and carrier phase wavelength respectively. This means, e.g., the noise for the GPS C/A code and P code are approximately 3 m and 0.3 m, respectively, and the noise for the carrier phase is about 0.003 m. When smoothing the code with the carrier phase, the C/A code noise might be reduced to approximately 0.6 m. In addition, the pseudorange noise also depends on the signal strength, which varies with the elevation angle. Finally, the pseudorange noise is generally larger in kinematic conditions as well. Take this into account, assuming that the carrier phase noise is $\sigma_{\varphi }=0.003$ m, the pseudorange noise is $\sigma_{P}=\left(0.3,0.6,3\right)$ m, the combined wavelength is larger than 4 m, and |i+j+k|≤2, the optimal geometry-free pseudorange minus phase linear combinations can be searched by setting i, j, and k within the range of [−10,10]. Table 2 and Table 3 list the first seven optimal pseudorange minus phase combinations for BDS and GPS, respectively. Columns 1 through 3 are coefficients of pseudorange minus phase combinations. Columns 4 and 5 are combined wavelength and ionospheric scale factors, respectively. Columns 6 through 8 are STDs of estimated combined cycle slip where $\sigma_{P}=0.3,0.6,\;\mathrm{and}\;3$ m, respectively.

Table 2 and Table 3 show that the listed pseudorange minus phase linear combinations are not affected as much by the pseudorange noise due to their larger wavelength. The STDs of estimated combined cycle slip are generally smaller than 0.2 cycles in the case of 0.3 m and 0.6 m pseudorange noise, which results in at least a 98.8% rounding success rate for cycle slip detection. The STDs can be less than 0.15 cycles in certain cases, which can result in at least a 99.9% rounding success rate. However, the STDs for all the pseudorange minus phase linear combinations are larger than 0.2 cycles in the case of 3 m pseudorange noise. For combinations with wavelengths that are less than 10 m, the impact of pseudorange noise can be significant, and the STDs can be larger than 0.3 cycles. In such cases, the rounding success rate can be relatively small.

It should be noted that the impact of ionospheric delay can be effectively reduced by using a second-order time difference of pseudorange minus phase combinations, but at the same time, the observation noise is significantly amplified. The impact of observation noise can be reduced by choosing pseudorange minus phase combinations with larger wavelengths. In the case of low pseudorange noise, combinations with small STDs can be derived. Contrary to this, the combined noise can be relatively large in the case of large pseudorange noise, which makes it unsuitable for cycle slip detection.

Table 4 and Table 5 list the optimal pseudorange minus phase combinations for BDS and GPS cycle slip detection and repair, respectively. Columns 1 through 3 list the coefficients of the first combination, columns 4 through 6 list the coefficients of the second combination, and columns 7 through 9 list the coefficients of the third combination. The final three columns are the minimum probability for cycle slip detection when $\sigma_{P}=0.3,0.6,\;\mathrm{and}\;3$ m, respectively. Table 4 and Table 5 show that the cycle slip detection success rates can reach up to 99.9% and 99.2% for BDS and GPS if the pseudorange noise is 0.3 m and 0.6 m, respectively. However, the highest cycle slip detection success rate is only approximately 95% if the pseudorange noise is 3 m. 

## 4. Data Tests and Analysis

### 4.1. Static Test

**Figure 2:**
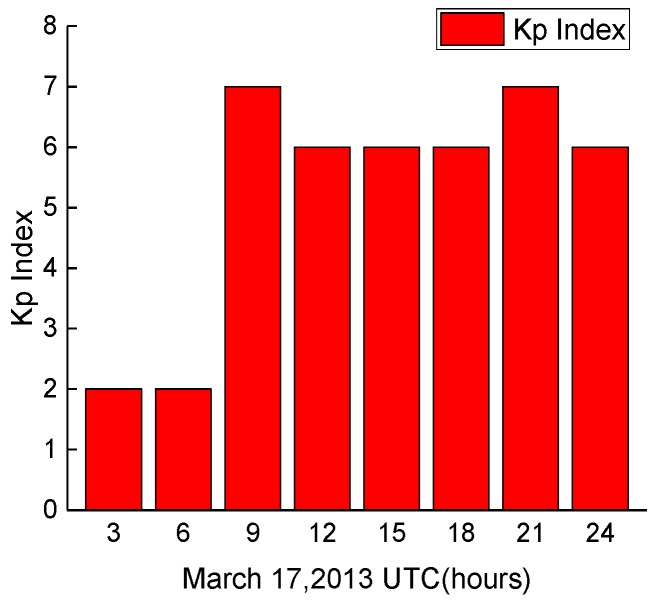
Geomagnetic Kp index from 17 March 2013.

According to [44], a moderate geomagnetic storm occurred in the middle of March 2013. Figure 2 shows the geomagnetic Kp index from 17 March 2013. The average Kp index is 5.25, which also indicates that the level of ionospheric activity was high. The Earth’s magnetic field was disturbed dramatically in the near-equatorial, mid-latitude, and auroral regions. Triple-frequency BDS/GPS observations collected from the multi-GNSS experiment (MGEX) station JFNG (30.52° N, 114.49° E)—which is in the area where the Total Election Content (TEC) changed significantly on 17 March 2013—were used to verify the performance of the proposed method. The observations were collected with a 30 s sampling interval using a Trimble NetR9 receiver, which was connected to a TRM59800 antenna. BDS C03, C09, C12 and GPS G01, G24, G25 were tested, and simulated cycle slips were previously added to their corresponding observations.

We first analyzed ionospheric delay variations. The first-order, between-epoch ionospheric delay variations can be estimated using $\dot{I}=f_{3}^{2}\left(\lambda_{1}{\dot{\varphi }}_{1}-\lambda_{3}{\dot{\varphi }}_{3}\right)/\left(f_{1}^{2}-f_{3}^{2}\right)$, and the second-order, between-epoch time-difference ionospheric delay variations can be calculated using ${\ddot{I}}_{i}={\dot{I}}_{i}-{\dot{I}}_{i-1}$.

**Figure 3:**
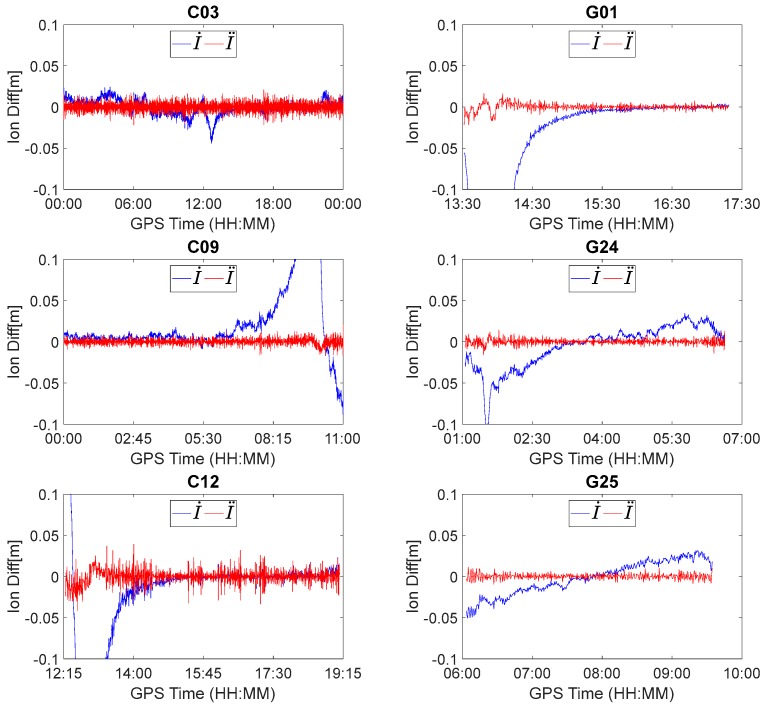
Between-epoch first-order and second-order ionospheric delay variations for C03, C09, C12, G01, G24, and G25.

Figure 3 displays the between-epoch, first-order and second-order ionospheric delay variations of BDS C03, C09, C12 and GPS G01, G24, G25. The first-order ionospheric delay variations for all the involved satellites, except for C03, were fairly significant and cannot be ignored due to the impact of the magnetic storm. This is reasonable considering that the signal propagation path of C03 in the ionosphere varies much more slowly than the other satellites due to its slower movement in the Geostationary Earth Orbit (GEO). However, when $\dot{I}$ is further differenced in time, the trend is removed and the second-order ionospheric delay variations (i.e., $\ddot{I}$) for all the involved satellites generally vary within ±2 cm. This indicates that the impact of ionospheric delay can be significantly reduced using the between-epoch, second-order, time-difference algorithm. Consequently, the rounding success rate for the estimated float combined cycle slips using the second-order time-difference algorithm can be significantly improved compared to the first-order time-difference algorithm.

According to Table 4 and Table 5, the optimal geometry-free pseudorange minus phase combinations were [−4, 1, 4], [−3, 6, −2], [4, −2, −3] for BDS and [−6, 1, 7], [3, 0, −4], [4, −8, 3] for GPS, respectively. To evaluate the effectiveness of the proposed method, two types of evenly distributed cycle slips were simulated. 

① The combined cycle slips satisfy the condition of $\left|i{\dot{N}}_{1}+j{\dot{N}}_{2}+k{\dot{N}}_{3}\right|\leq 1$ to verify the performance in identifying small combined cycle slips. 

② The cycle slips for each of the original carrier phase observations were defined as 1 cycle to verify the ability of identifying small cycle slips. 

**Figure 4:**
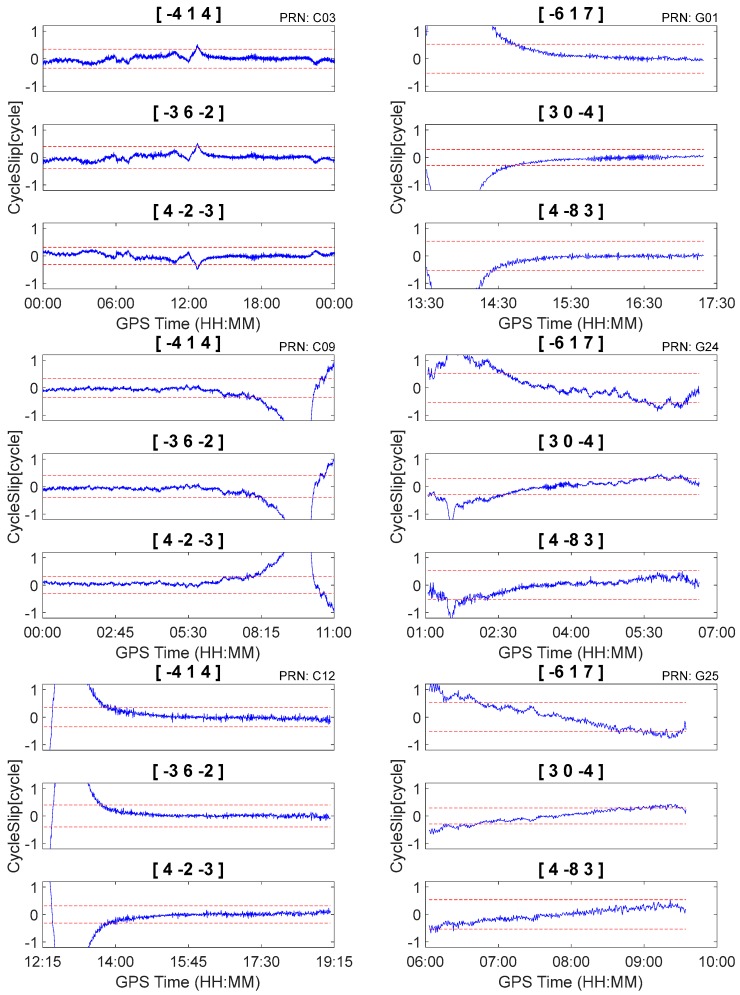
Cycle slip detection by first-order between-epoch difference pseudorange minus phase combination for C03, C09, C12, G01, G24, and G25.

First, we used three independent, geometry-free, pseudorange minus phase combinations to detect and repair the cycle slips on the original carrier phase observations using Equation (6) and ignored the between-epoch ionospheric delay variations. According to Equation (6), when $\dot{I}$ is sufficiently large, misjudgment may occur even if no cycle slips occur on the original carrier phase observations (i.e., ${\dot{N}}_{ijk}=0$(${\dot{N}}_{1}={\dot{N}}_{2}={\dot{N}}_{3}=0$)). Consequently, the performance will be reduced under conditions of high ionospheric activity. The cycle slip detection results for raw observations are shown in Figure 4. It is apparent that misjudgments occur in all cases due to the presence of relatively large between-epoch ionospheric delay variations.

**Figure 5:**
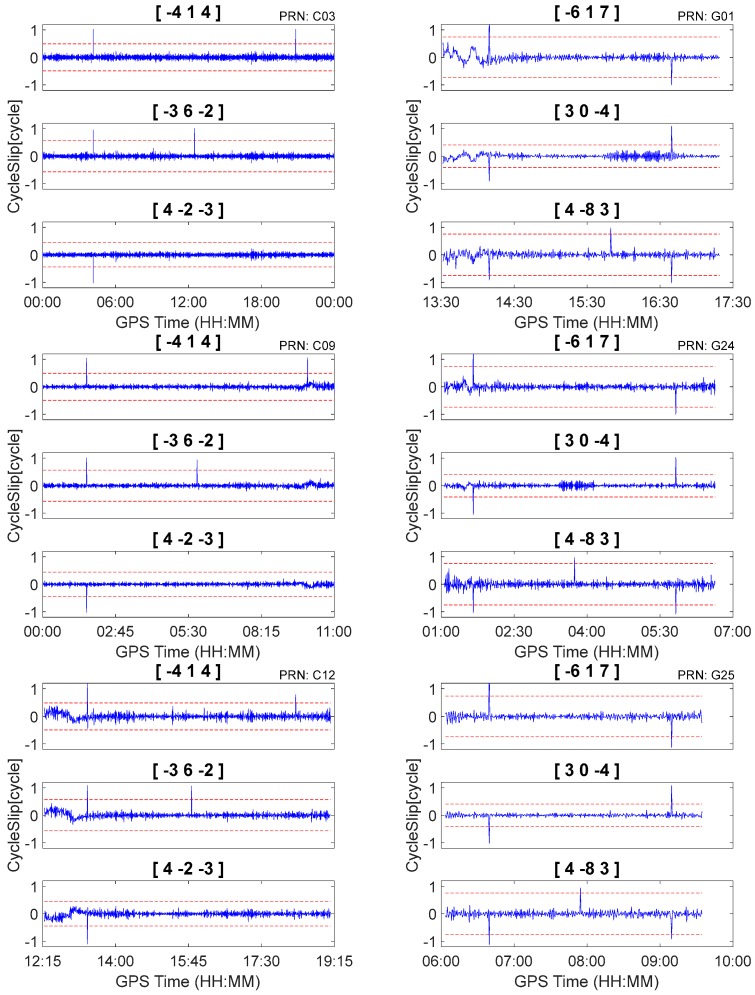
Cycle slip detection by second-order between-epoch difference pseudorange minus phase combination for C03, C09, C12, G01, G24, and G25.

Second, the proposed method was tested with data that were inserted with simulated cycle slips. Cycle slip detection and repair results are shown in Figure 5 and Table 6. It is apparent that the proposed method can correctly detect and repair simulated cycle slips that are as small as 1 cycle. Moreover, there were no insensitive cycle slip pairs that occurred in response to the proposed method. For example, although BDS cycle slips pair (5, 4, 4) cannot be identified by geometry-free pseudorange minus phase combinations [−4, 1, 4] and [4, −2, −3], it was obviously identified by [−3, 6, 2]. Consequently, it can still be uniquely determined by the three independent cycle slip detection combinations. Finally, the simulated cycle slips can be correctly identified even under conditions of high levels of ionospheric activity. Using C09 for example, as shown in Figure 5, the between-epoch ionospheric delay variations can exceed 0.1 m from GPST 9:00 to 10:00. However, after applying the second-order time difference, the ionospheric variations are close to zero, and the simulated cycles-slips at GPST 09:59:30 can be correctly detected and repaired (as shown in Figure 5 and Table 6).

### 4.2. Kinematic Test

In this section, BDS observations from the car-driven experiment and GPS observations from the airborne experiment are used to further evaluate the real-time cycle slip detection and repair performance of the proposed method under kinematic conditions. The ionosphere is quiet for both the car-driven experiment and airborne experiment, and the simulated cycle slips are previously inserted to observations. To reduce the impact of relatively large pseudorange noise in the kinematic environments, geometry-free pseudorange minus phase combinations with relatively large wavelengths were chosen. Consequently, according to Table 2 through Table 5, the chosen BDS cycle slip detection combinations were [−4, 1, 4], [−3, 6, −2], and [4, −2, −3], which had combined wavelengths of 8.14 m, 13.321 m, and 12.211 m, respectively. The chosen GPS cycle slip detection combinations were [−6, 1, 7], [3, 0, −4], and [4, −8, 3], which had combined wavelengths of 29.305 m, 14.653 m, and 29.305 m, respectively. 

#### 4.2.1. Kinematic Test for BDS

**Figure 6:**
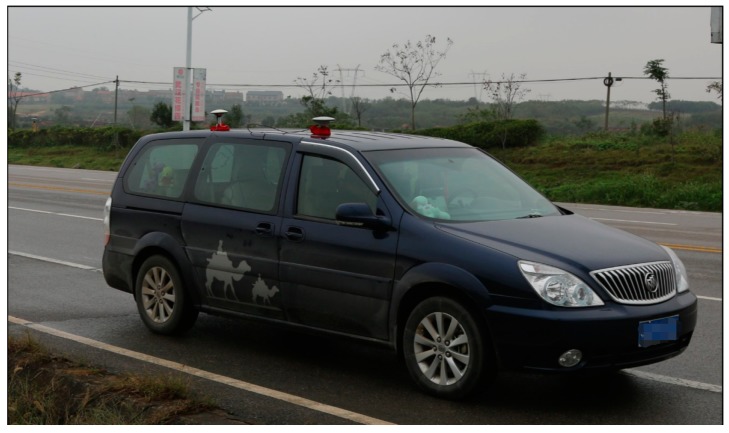
Observational environments and GNSS antennas in the car-driven experiment.

**Figure 7:**
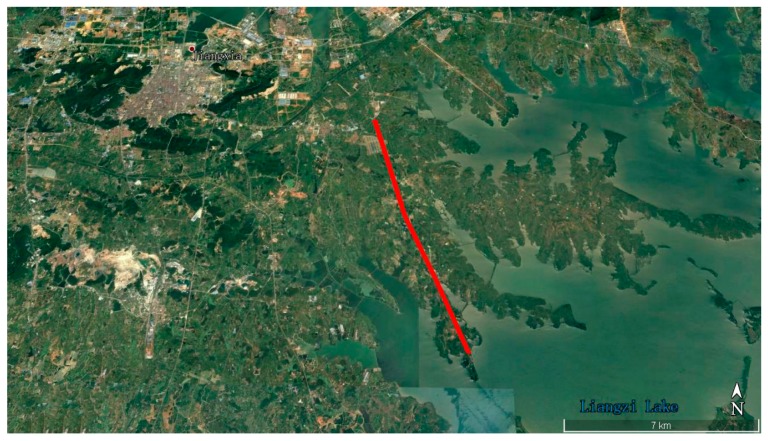
Trajectory of the car.

**Figure 8:**
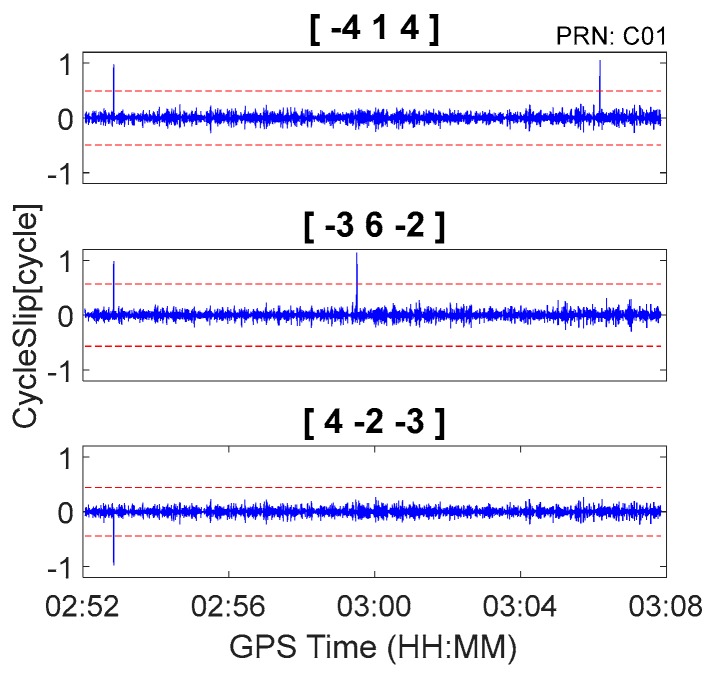
Cycle slip detection for C01 with sampling interval of 0.5 s.

**Figure 9:**
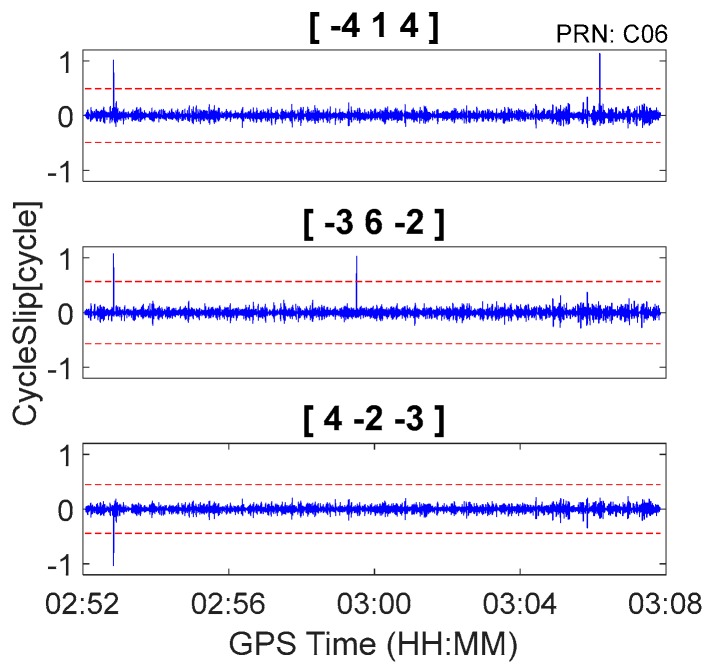
Cycle slip detection for C06 with sampling interval of 0.5 s.

**Figure 10:**
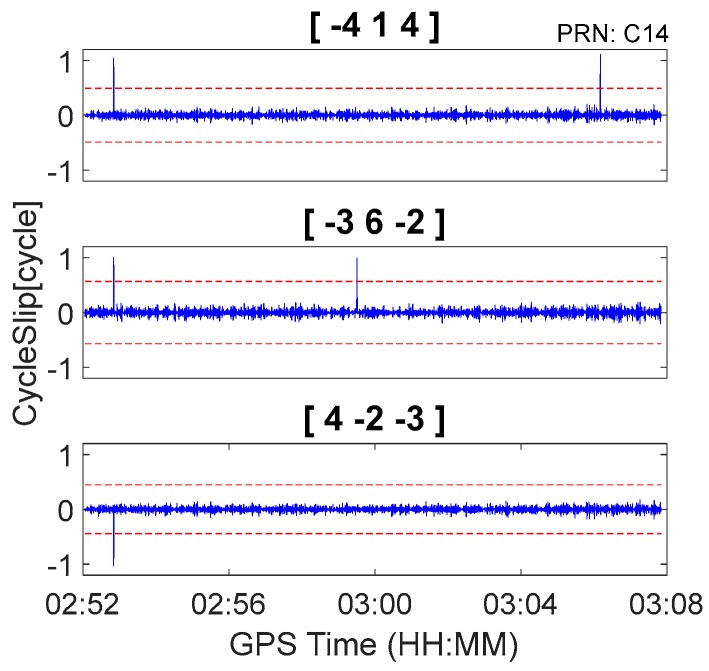
Cycle slip detection for C14 with sampling interval of 0.5 s.

The BDS kinematic data were collected from the car-driven experiment conducted in Wuhan on 27 October 2016. The sampling interval was 0.5 s. The type of GNSS receiver and antenna that were used in the experiment were a ComNav M300 receiver and a NovAtel 704-WB antenna. The observational environments and GNSS antennas in car-driven experiment are shown in Figure 6. The trajectory of the car is shown in Figure 7. The speed of car was approximately 50 km/h. Observations from C01, C06, and C14 were analyzed to verify the cycle slip detection and repair results of the proposed method and are shown in Figure 8, Figure 9 and Figure 10 and in Table 7.

#### 4.2.2. Kinematic Test for GPS

**Figure 11:**
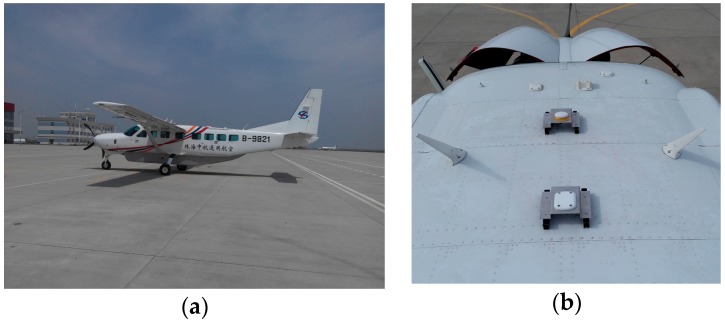
(**a**) Airplane used in the airborne experiment; (**b**) GNSS antenna used in the airborne experiment.

**Figure 12:**
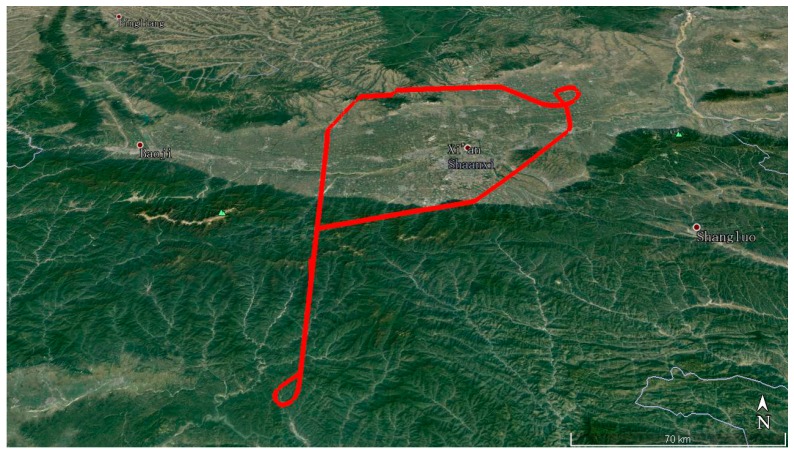
Trajectory of the airplane.

**Figure 13:**
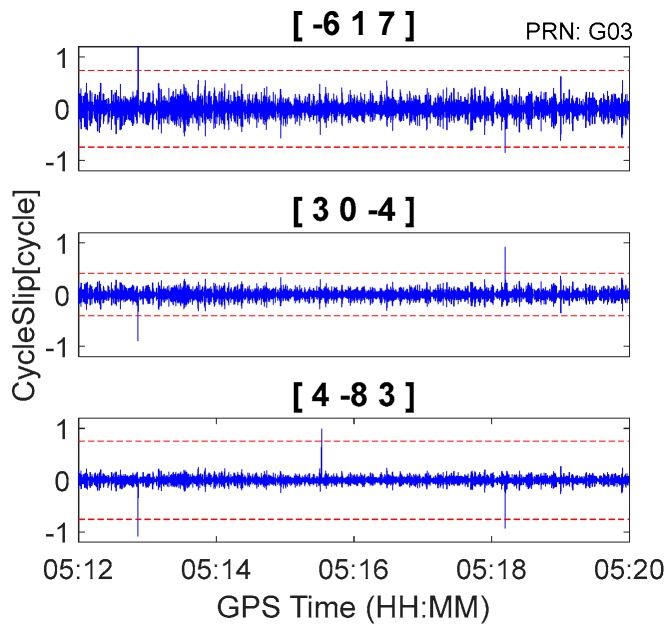
Cycle slip detection for G03 with sampling interval of 0.2 s.

**Figure 14:**
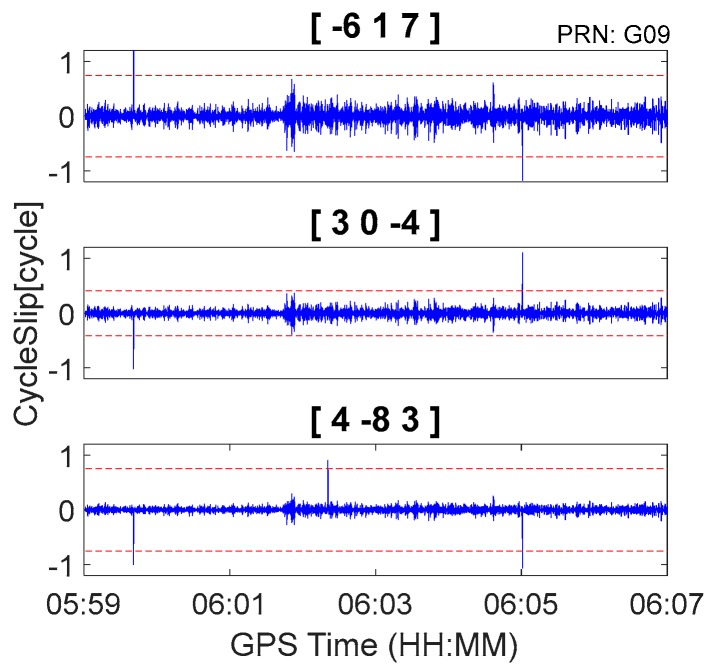
Cycle slip detection for G09 with sampling interval of 0.2 s.

**Figure 15:**
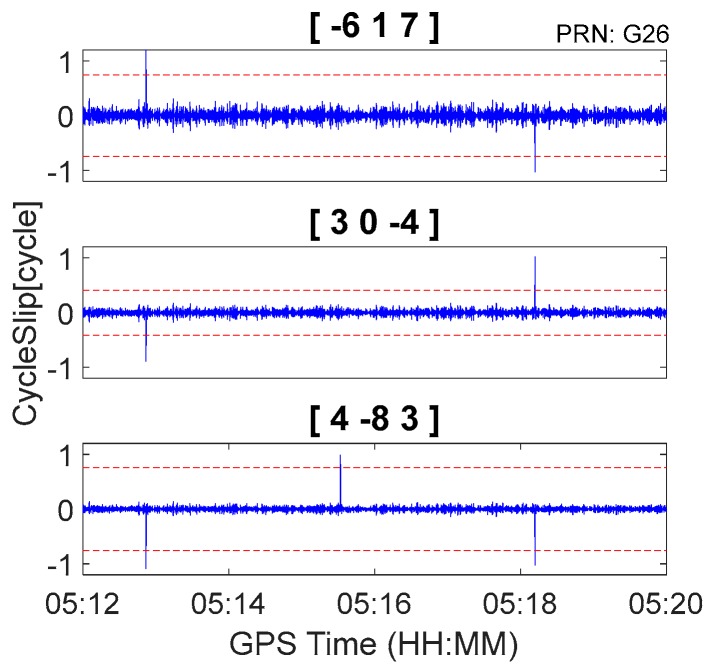
Cycle slip detection for G26 with sampling interval of 0.2 s.

The GPS kinematic data were collected from the airborne experiment conducted at Xi’an in China on 24 April 2015. The sampling interval was 0.2 s. The type of GNSS receiver and antenna that were used in the experiment were a NovAtel ProPak6 receiver and an Antcom aviation GNSS antenna (743GNSSA-XT-1). The airplane and antenna used in the airborne experiment are shown in Figure 11. The trajectory of the airplane is shown in Figure 12. The speed of airplane was approximately 300 km/h. Observations from G03, G09, and G26 were analyzed to verify the cycle slip detection and repair results of the proposed method and are shown in Figure 13, Figure 14 and Figure 15 and in Table 8.

#### 4.2.3. Analysis of Results

It is apparent in Figure 8, Figure 9 and Figure 10 and Figure 13, Figure 14 and Figure 15 that the proposed method can also be applied to process kinematic observations to effectively identify and repair cycle slips that are as small as 1 cycle.

The pseudorange noise observed during the kinematic experiments is generally larger than that observed during the static experiments. According to Equation (7), the impact of pseudorange noise decreases when divided by a larger combined wavelength. Optimal combinations with larger wavelengths can be constructed by using triple-frequency observations compared to dual-frequency observations. As such, the availability of triple-frequency observations plays an important role in cycle slip detection and repair, especially for preprocessing observations collected from kinematic experiments.

### 4.3. Summary of Static and Kinematic Test Results

In summary, the proposed method can detect and repair as small as 1 cycle slip for both static and kinematic observations. Moreover, the cycle slip detection possibility is improved under high ionospheric activity, and there are no insensitive cycle slips existing in our method.

## 5. Conclusions

In this paper, we present a new real-time cycle slip detection and repair method for undifferenced GPS/BDS triple-frequency observations collected by a single receiver under conditions of high ionospheric activity. In the approach, the cycle slips on the original triple-frequency carrier phase observations were correctly and uniquely determined by applying the second-order time difference algorithm to three linearly independent geometry-free pseudorange minus phase combinations. The proposed approach has multiple advantages. First, no insensitive cycle slips exist in our method. Second, the impact of ionospheric delay variations can be significantly reduced by using the second-order time-difference algorithm even if the between-epoch ionospheric delay varies rapidly, resulting in an improved cycle slip rounding success rate. Finally, triple-frequency carrier phase and pseudorange observations of only three consecutive epochs are required for data processing. Therefore, the method enables the implementation of high-efficiency cycle slip detection and repair in real time.

The performance of the method was verified by both static and kinematic tests. It was shown that the method can detect and correctly repair cycle slips as small as 1 cycle under conditions of high ionospheric activity. Additionally, the method is effective in processing kinematic data. However, the performance of the method may be influenced by the pseudorange noise level. The cycle slip combinations can be determined with a rounding success rate of more than 99% in the case of the 0.3 m and 0.6 m pseudorange noise, whereas the success rate is only approximately 95% in the case of the 3 m pseudorange noise. These results indicate that the cycle slip detection performance of the presented method is reduced when pseudorange noise is particularly large (e.g., 3 m or larger).

## Figures and Tables

**Table 1:** Rounding success rates for $\sigma_{{\hat{\dot{N}}}_{ijk}}=0.15,0.2,0.25$.

$\sigma_{{\hat{\dot{N}}}_{ijk}}$ (Cycles)	Rounding Success Rate
0.15	99.9%
0.2	98.8%
0.25	95.5%

**Table 2:** BeiDou Navigation Satellite System (BDS) geometry-free pseudorange minus phase linear combinations.

i	j	k	λ(m)	K	STD (Cycles)		
					$\sigma_{P}=0.3\;m$	$\sigma_{P}=0.6\;m$	$\sigma_{P}=3\;m$
−4	1	4	8.14	11.71	0.123	0.143	0.441
−3	6	−2	13.321	12.071	0.142	0.149	0.295
−1	−5	6	20.932	−0.362	0.158	0.161	0.228
0	−1	1	4.884	−0.04	0.076	0.145	0.71
4	−2	−3	12.211	−11.75	0.111	0.122	0.303
5	3	−9	29.305	−11.388	0.215	0.216	0.245
7	−8	−1	146.526	−23.821	0.214	0.214	0.215

STD, standard deviation.

**Table 3:** Global Positioning System (GPS) geometry-free pseudorange minus phase linear combinations.

i	j	k	λ(m)	K	STD (Cycles)		
					$\sigma_{P}=0.3\;m$	$\sigma_{P}=0.6\;m$	$\sigma_{P}=3\;m$
−6	1	7	29.305	24.525	0.186	0.187	0.22
−3	1	3	9.768	12.242	0.094	0.112	0.365
−1	8	−7	29.305	−0.513	0.214	0.215	0.244
0	1	−1	5.861	−0.041	0.066	0.122	0.592
3	0	−4	14.653	−12.283	0.103	0.111	0.257
4	−8	3	29.305	−11.77	0.189	0.19	0.223
7	−8	−1	9.768	−24.052	0.216	0.225	0.414

**Table 4:** Pseudorange minus phase combinations for BDS cycle slip detection and repair.

$i_{1}$	$j_{1}$	$k_{1}$	$i_{2}$	$j_{2}$	$k_{2}$	$i_{3}$	$j_{3}$	$k_{3}$	$P_{\sigma_{P}=0.3\;m}$	$P_{\sigma_{P}=0.6\;m}$	$P_{\sigma_{P}=3\;m}$
−4	1	4	−3	6	−2	0	−1	1	99.96%	99.92%	51.88%
−4	1	4	−3	6	−2	4	−2	−3	99.96%	99.92%	74.33%
−3	6	−2	0	−1	1	4	−2	−3	99.96%	99.92%	51.88%
−1	−5	6	5	3	−9	7	−8	−1	98.01%	97.95%	95.88%

**Table 5:** Pseudorange minus phase combinations for GPS cycle slip detection and repair.

$i_{1}$	$j_{1}$	$k_{1}$	$i_{2}$	$j_{2}$	$k_{2}$	$i_{3}$	$j_{3}$	$k_{3}$	$P_{\sigma_{P}=0.3\;m}$	$P_{\sigma_{P}=0.6\;m}$	$P_{\sigma_{P}=3\;m}$
−6	1	7	3	0	−4	4	−8	3	99.18%	99.14%	94.86%
−3	1	3	3	0	−4	4	−8	3	99.18%	99.14%	82.91%
−6	1	7	−3	1	3	4	−8	3	99.18%	99.14%	82.91%
−6	1	7	−1	8	−7	4	−8	3	98.06%	98.00%	95.95%

**Table 6:** Cycle slip detection and repair results.

PRN	Time (HH:MM:SS)	Simulated Cycle Slip	${\hat{\dot{N}}}_{i_{1}j_{1}k_{1}}$	${\hat{\dot{N}}}_{i_{2}j_{2}k_{2}}$	${\hat{\dot{N}}}_{i_{3}j_{3}k_{3}}$	Computed Integer Cycle Slip
C03	04:09:30	(1,1,1)	1.02	0.96	−1.02	(1,1,1)
	12:29:30	(5,4,4)	−0.02	1.02	−0.01	(5,4,4)
	20:49:30	(22,17,18)	1.02	0.01	−0.06	(22,17,18)
C09	01:39:30	(1,1,1)	1.04	1.01	−1.03	(1,1,1)
	05:49:30	(5,4,4)	−0.03	0.94	0.01	(5,4,4)
	09:59:30	(22,17,18)	1.05	0.09	−0.01	(22,17,18)
C12	13:19:30	(1,1,1)	1.18	1.08	−1.10	(1,1,1)
	15:49:30	(5,4,4)	0.04	1.07	−0.06	(5,4,4)
	18:19:30	(22,17,18)	0.80	−0.04	0.08	(22,17,18)
G01	14:09:30	(1,1,1)	1.77	−0.91	−0.91	(1,1,1)
	15:49:30	(5,4,4)	0.08	−0.20	0.98	(5,4,4)
	16:39:30	(22,17,18)	−1.01	1.03	−1.08	(22,17,18)
G24	01:39:30	(1,1,1)	2.09	−1.06	−1.04	(1,1,1)
	03:44:30	(4,3,3)	−0.02	0.01	0.97	(4,3,3)
	05:49:30	(23,18,17)	−1.01	1.03	−1.08	(23,18,17)
G25	06:39:30	(1,1,1)	1.96	−1.03	−1.13	(1,1,1)
	07:54:30	(4,3,3)	0.07	−0.08	0.95	(4,3,3)
	09:09:30	(23,18,17)	−1.14	1.08	−0.91	(23,18,17)

PRN, pseudorandom noise code.

**Table 7:** Cycle slip detection and repair results.

PRN	Time (HH:MM:SS)	Simulated Cycle Slip	${\hat{\dot{N}}}_{i_{1}j_{1}k_{1}}$	${\hat{\dot{N}}}_{i_{2}j_{2}k_{2}}$	${\hat{\dot{N}}}_{i_{3}j_{3}k_{3}}$	Computed Integer Cycle Slip
C01	02:53:40	(1,1,1)	0.97	0.98	−0.97	(1,1,1)
	03:00:20	(5,4,4)	−0.04	1.14	0.02	(5,4,4)
	03:07:00	(22,17,18)	1.05	0.02	−0.03	(22,17,18)
C06	02:53:40	(1,1,1)	1.01	1.08	−1.03	(1,1,1)
	03:00:20	(5,4,4)	−0.05	1.03	0.04	(5,4,4)
	03:07:00	(22,17,18)	1.13	0.04	−0.17	(22,17,18)
C14	02:53:40	(1,1,1)	1.03	1.00	−1.03	(1,1,1)
	03:00:20	(5,4,4)	−0.01	1.00	0.00	(5,4,4)
	03:07:00	(22,17,18)	1.11	−0.04	−0.08	(22,17,18)

**Table 8:** Cycle slip detection and repair results.

PRN	Time (HH:MM:SS)	Simulated Cycle Slip	${\hat{\dot{N}}}_{i_{1}j_{1}k_{1}}$	${\hat{\dot{N}}}_{i_{2}j_{2}k_{2}}$	${\hat{\dot{N}}}_{i_{3}j_{3}k_{3}}$	Computed Integer Cycle Slip
G03	05:13:51	(1,1,1)	1.81	−0.89	−1.08	(1,1,1)
	05:16:31	(4,3,3)	−0.06	0.03	0.99	(4,3,3)
	05:19:11	(23,18,17)	−0.85	0.91	−0.92	(23,18,17)
G09	06:00:31	(1,1,1)	2.01	−1.01	−1.00	(1,1,1)
	06:03:11	(4,3,3)	−0.21	0.12	0.90	(4,3,3)
	06:05:51	(23,18,17)	−1.17	1.10	−1.06	(23,18,17)
G26	05:13:51	(1,1,1)	1.80	−0.88	−1.09	(1,1,1)
	05:16:31	(4,3,3)	0.01	0.00	0.99	(4,3,3)
	05:19:11	(23,18,17)	−1.03	1.02	−1.02	(23,18,17)
